# Cancer Patient-Derived Cell-Based Models: Applications and Challenges in Functional Precision Medicine

**DOI:** 10.3390/life14091142

**Published:** 2024-09-10

**Authors:** Jelena Dinić, Sofija Jovanović Stojanov, Miodrag Dragoj, Marija Grozdanić, Ana Podolski-Renić, Milica Pešić

**Affiliations:** Department of Neurobiology, Institute for Biological Research “Siniša Stanković”—National Institute of the Republic of Serbia, University of Belgrade, Bulevar Despota Stefana 142, 11108 Belgrade, Serbia; jelena.dinic@ibiss.bg.ac.rs (J.D.); sofija.jovanovic@ibiss.bg.ac.rs (S.J.S.); miodrag.dragoj@ibiss.bg.ac.rs (M.D.); marija.grozdanic@ibiss.bg.ac.rs (M.G.); ana.podolski@ibiss.bg.ac.rs (A.P.-R.)

**Keywords:** anticancer drugs, targeted therapy, functional assays, drug resistance, functional precision medicine

## Abstract

The field of oncology has witnessed remarkable progress in personalized cancer therapy. Functional precision medicine has emerged as a promising avenue for achieving superior treatment outcomes by integrating omics profiling and sensitivity testing of patient-derived cancer cells. This review paper provides an in-depth analysis of the evolution of cancer-directed drugs, resistance mechanisms, and the role of functional precision medicine platforms in revolutionizing individualized treatment strategies. Using two-dimensional (2D) and three-dimensional (3D) cell cultures, patient-derived xenograft (PDX) models, and advanced functional assays has significantly improved our understanding of tumor behavior and drug response. This progress will lead to identifying more effective treatments for more patients. Considering the limited eligibility of patients based on a genome-targeted approach for receiving targeted therapy, functional precision medicine provides unprecedented opportunities for customizing medical interventions according to individual patient traits and individual drug responses. This review delineates the current landscape, explores limitations, and presents future perspectives to inspire ongoing advancements in functional precision medicine for personalized cancer therapy.

## 1. Introduction

Cancer continues to be one of the leading causes of death worldwide, and it requires ongoing advancements in treatment methods. Throughout the years, cancer therapy has undergone notable progress as a result of multidisciplinary efforts. Today, cancer research continues to focus on the development of new therapeutic approaches, including the study of tumor evolution and the use of liquid biopsies to monitor clonal evolution and guide personalized therapy [1]. The development of next-generation sequencing (NGS) technologies has enabled the rapid and cost-effective analysis of a large number of genes, allowing more accurate diagnosis and treatment of cancer. In addition, the use of artificial intelligence and machine learning algorithms is being explored to improve the accuracy of cancer diagnosis and treatment.

Personalized therapy has revolutionized the field of oncology by greatly impacting cancer drug treatments. Novel targeted therapeutics, such as tyrosine kinase inhibitors (TKIs) and monoclonal antibodies, have led to more effective treatments and better outcomes. The selectivity and specificity of novel drugs have increased survival rates, reduced the side effects often associated with conventional chemotherapy and radiation, and improved patients’ quality of life during and after treatment. Immune checkpoint inhibitors have revolutionized the treatment of various cancers by stimulating the body’s own immune response to attack and kill cancer cells. Advances in omics approaches have enabled the identification of biomarkers that predict how patients will respond to specific treatments and facilitate the selection of the most effective therapies tailored to the individual patient [2]. Comprehensive genetic profiles of tumors help identify actionable mutations and enable oncologists to select the most appropriate targeted therapies. In addition, the issue of drug resistance has been tackled by personalized therapies, using combination treatments, and creating advanced inhibitors to maintain long-term effectiveness [3,4,5].

Having initially been successful in hematological cancers, targeted therapies are now being applied to a wide range of solid cancers, further enhancing their impact. Artificial intelligence and big data analytics are increasingly being used to predict treatment responses and outcomes and to refine and optimize personalized treatment plans [2]. Importantly, patients are now better informed about their treatment options and can participate in the decision-making process, resulting in more personalized care.

Despite its potential, personalized therapy still faces various challenges. These include the limited eligibility of cancer patients to receive targeted drugs and the complexity of tumor heterogeneity. Overcoming these challenges is crucial for the widespread application of personalized therapy. As a result, new efforts are being made to conduct functional screenings of drugs and their combinations for each patient to find the best treatment option. What is surprising to scientists is that some eligible patients do not respond well to specific targeted therapies. In contrast, non-eligible patients can respond positively to targeted therapies for which they do not possess the necessary biomarkers.

Functional precision medicine (FPM) combines omics profiling with sensitivity testing of patient-derived cancer cells to identify effective treatments. This approach has shown significant value in personalized therapy by maintaining primary tumor features, accurately replicating patient responses to treatments, distinguishing good responders from bad responders, and identifying treatment options for a higher percentage of patients than genotyping alone [6]. The field of FPM is dedicated to customizing medical treatment based on individual patient traits. It utilizes two-dimensional (2D) and three-dimensional (3D) cell cultures, patient-derived xenograft (PDX) models, and cancer-on-a-chip systems to investigate patient-specific reactions to therapeutic interventions. These models enable the development of personalized treatment approaches by simulating the patient’s cancer characteristics, offering insights into tumor behavior and drug response. Functional assays, such as cell viability and cytotoxicity assays, gene and protein expression analysis, and high-content screening, are pivotal in examining cell behavior, drug response, and tissue modeling in 2D and 3D patient-derived cultures.

## 2. The Evolution of Cancer-Directed Drugs

### 2.1. Conventional Therapies

Before the introduction of targeted therapies, cancer treatment was mainly based on non-specific methods such as chemotherapy and radiotherapy [7,8]. These traditional approaches have played a central role in cancer treatment for decades. However, conventional cancer therapy has undergone significant changes over the years due to advances in molecular biology and omics approaches [2]. In the early 20th century, radiation therapy emerged as a treatment option for cancer, offering a non-invasive and relatively safe way to treat localized tumors, beginning a new era in cancer treatment. By the 1940s, the first chemotherapeutic agents are developed, marking the beginning of modern oncology. These early drugs were derived from natural sources such as plants and microorganisms and were used to treat various types of cancer. Although these early drugs were not very effective, they laid the foundation for future advances. Despite their lack of specificity, chemotherapy and radiotherapy are still central to cancer treatment and often lead to significant side effects and variable effectiveness.

Chemotherapy uses drugs that target and kill rapidly dividing cells—a characteristic feature of cancer cells. However, because chemotherapy drugs are not selective, they also attack healthy cells that divide rapidly, such as those in the bone marrow, digestive tract, and hair follicles, leading to side effects such as immunosuppression, gastrointestinal problems, and hair loss [7,9]. Despite their limitations, these traditional therapies are essential in cancer treatment, especially when surgical options are limited or the cancer has spread beyond the primary site. They form the basis of cancer treatment protocols and have helped to improve survival rates for many types of cancer [8]. Chemotherapeutic agents are categorized according to their mechanisms of action and specific targets in the cell cycle. These include alkylating agents such as cyclophosphamide and cisplatin, antimetabolites such as methotrexate and 5-fluorouracil (5-FU), anti-tumor antibiotics such as doxorubicin and bleomycin, mitosis inhibitors such as paclitaxel and vincristine, and topoisomerase inhibitors such as etoposide and irinotecan [8]. Radiotherapy uses high-energy radiation to damage the DNA of cancer cells, which ultimately leads to cell death [10]. Although radiotherapy is more localized compared to chemotherapy, it can still affect surrounding healthy tissue and cause side effects such as fatigue, skin reactions, and damage to nearby organs. Radiotherapy is supported by radiosensitizers such as cisplatin and 5-FU, which make the cancer cells more susceptible to radiation, and radioprotectors such as amifostine, which protect normal tissue from the side effects of radiation [11]. The combination of chemotherapy and radiation therapy, known as chemoradiation, has been shown to be effective in treating various types of cancer, as it maximizes the tumor-killing effect while minimizing damage to healthy tissue. Drugs such as cisplatin, 5-FU, and paclitaxel are often used in combination with radiation to improve outcomes in cancers such as head and neck cancer, endometrial cancer, and lung cancer [12].

### 2.2. Targeted Therapies

In the 1980s, targeted therapy became a reality with the development of specific drugs for molecular targets involved in neoplastic processes [2]. This was made possible by the discovery of the molecular mechanisms underlying the development and progression of cancer. Targeted therapies have been developed to specifically destroy cancer cells while minimizing damage to healthy cells, which has led to an improvement in survival rates and quality of life for cancer patients. Thanks to advances in DNA sequencing, genomics, transcriptomics, and proteomics in the 1990s, the first effective drugs for targeted therapies became available [2]. These technologies enabled the identification of new molecular targets and the development of drugs specifically aimed at these targets.

In the early 21st century, genetic engineering studies led to the introduction of monoclonal antibodies and immune checkpoint inhibitors for the treatment of advanced or metastatic tumors for which there were previously no effective treatment options.

Over the past two decades, the development of targeted therapies has revolutionized cancer treatment by providing more precise and personalized options. The focus shifted to specific molecular targets associated with cancer growth and progression, forming the basis for the significant progress in cancer drug development.

Current challenges in cancer treatment include the absence of unique biomarkers for individual cancer patients. Only 13% of cancer patients meet the criteria for targeted therapy [13]. However, among eligible patients, the response rate to targeted therapy ranges from 60% to 80%, as indicated in Table 1. Consequently, the overall efficacy of targeted therapy in the general population of cancer patients is approximately 7%. The clinical trials that led to the approval of targeted therapeutics primarily focused on progression-free survival (PFS), with limited reporting on overall survival (OS) [13,14,15,16,17,18,19,20,21,22,23,24,25,26,27,28,29,30,31,32,33,34,35,36,37,38,39,40,41,42,43,44,45,46,47,48,49,50,51,52,53,54,55,56,57,58,59,60,61,62,63,64,65,66,67,68,69,70,71,72,73,74,75,76,77,78,79,80,81,82,83] (Table 1). The true benefits of targeted therapy are expected to become clearer in the long term.

#### 2.2.1. Monoclonal Antibodies

Monoclonal antibodies (mAbs) represent a groundbreaking advance in targeted therapy, offering precision and effectiveness in the treatment of various types of cancer. The mechanism of action of mAbs involves their recognition of antigens on cancer cells and their high-specificity binding to these antigens, resulting in the marking of the cells for destruction by the immune system or the disruption of vital cellular processes [84]. This targeted approach not only reduces collateral damage to healthy cells but also increases the overall therapeutic efficacy of treatments. Moreover, mAbs can be engineered to deliver cytotoxic agents, radioactive isotopes, or immunostimulatory molecules directly to the tumor, enhancing their therapeutic effect while reducing systemic toxicity. Rituximab, a pioneering mAb approved in 1997, ushered in a new era in oncology by targeting CD20 antigens on B cells in patients with non-Hodgkin’s lymphoma [85]. Its success has enabled the development of a variety of mAbs tailored to target specific cancers and their unique biomarkers. One notable example is trastuzumab, which was developed to combat HER2-positive breast cancer [86]. By selectively binding to the HER2 receptor, trastuzumab inhibits signaling pathways that promote cancer growth, leading to better treatment outcomes for patients.

#### 2.2.2. Immune Checkpoint Inhibitors

In recent years, the development of immune checkpoint inhibitors, a type of mAb that enables the immune system to recognize and attack cancer cells, has further expanded the scope of immunotherapy in cancer treatment [87,88]. Pembrolizumab and nivolumab are examples that have achieved success in various cancers, including melanoma, lung cancer, and bladder cancer [89]. These drugs inhibit proteins such as programmed cell death protein-1 (PD-1) and cytotoxic T-lymphocyte-associated protein 4 (CTLA-4), which are used by cancer cells to evade immune recognition.

#### 2.2.3. Tyrosine Kinase Inhibitors

Tyrosine kinase inhibitors (TKIs) are an important class of targeted therapies that have revolutionized cancer treatment by targeting the abnormal activity of tyrosine kinases, key enzymes involved in the dysregulated signaling pathways that drive cancer growth and progression [90,91]. One of the main advantages of TKIs is their ability to selectively block specific tyrosine kinases, thereby disrupting critical signaling cascades that promote cancer cell proliferation, survival, and metastasis [91]. By targeting these pathways, TKIs offer a more precise and tailored treatment approach that minimizes damage to healthy cells and tissues compared to conventional therapies.

Imatinib was an important milestone in oncology when it was approved for chronic myeloid leukemia (CML) in 2001 [90]. Its success in effectively inhibiting the BCR-ABL fusion protein, a hallmark of CML, led to significant improvements in patient outcomes and survival rates. Other BCR-ABL inhibitors, such as bosutinib, dasatinib, nilotinib, and ponatinib, have also been shown to be effective in CML and acute lymphoblastic leukemia [91]. TKIs have demonstrated efficacy not only in hematologic malignancies such as CML but also in solid tumors such as lung cancer, breast cancer, gastrointestinal stromal tumors, and renal cell carcinoma. The success of imatinib led to the discovery and approval of subsequent generations of TKIs. These newer inhibitors target different tyrosine kinase receptors that play a role in different types of cancer and are characterized by improved efficacy, selectivity, and reduced off-target effects. Erlotinib and gefitinib, for example, have significantly expanded the therapeutic landscape by specifically targeting mutations of the epidermal growth factor receptor (EGFR) in non-small cell lung cancer (NSCLC) [91,92]. Other EGFR inhibitors such as afatinib, dacomitinib, lapatinib, neratinib, osimertinib, and vandetanib are used in various cancers such as NSCLC, small cell lung cancer, and breast cancer and target EGFR mutations. Other important TKIs include ALK inhibitors such as alectinib, brigatinib, ceritinib, crizotinib, entrectinib, and lorlatinib, which are mainly used in ALK-positive NSCLC [92]. FLT3 inhibitors such as gilteritinib and midostaurin target acute myeloid leukemia with FLT3 mutations, while FGFR inhibitors such as erdafitinib are used for urothelial carcinoma [93,94]. NTRK inhibitors such as larotrectinib are effective in solid tumors with NTRK gene fusions [95]. VEGFR inhibitors such as axitinib, carbozantinib, lenvatinib, pazopanib, regorafenib, sorafenib, and sunitinib are effective against various types of cancer by inhibiting vascular endothelial growth factor receptors [91,96]. BRAF inhibitors such as dabrafenib, encorafenib, and vemurafenib focus on melanoma and NSCLC with BRAF mutations [97], while Bruton tyrosine kinase inhibitors such as acalabrutinib and ibrutinib are used for mantle cell lymphoma and chronic lymphocytic leukemia, among others [98]. MEK inhibitors such as binimetinib, cobimetinib, and trametinib are aimed at melanomas and NSCLC with BRAF mutations [99]. Finally, CDK4/6 inhibitors such as abemaciclib, palbociclib, and ribociclib are used for hormone receptor-positive breast cancer [100]. These TKIs have significantly improved the precision and efficacy of cancer therapies by focusing on specific genetic mutations and molecular targets, improving patient outcomes, and reducing side effects compared to conventional chemotherapy and radiotherapy.

In addition to their efficacy as a single therapy, TKIs have also been shown to be valuable in combination with other targeted therapies, chemotherapy, immunotherapy, or radiotherapy, highlighting their versatility and potential for synergistic effects in overcoming treatment resistance and improving overall outcomes for cancer patients [90]. Ongoing research and development of TKIs, including efforts to overcome resistance mechanisms, identify new targets, and refine treatment strategies through biomarker-driven approaches, promise further advances in personalized cancer therapy.

## 3. Resistance to Cancer-Directed Drugs

The effectiveness of chemotherapy in patients is often reduced by the development of drug resistance. Acquired resistance is especially problematic, as cancer cells can become resistant not only to the specific anticancer drug being used but also to other drugs with different structures and mechanisms of action [101]. Drug resistance can occur at various levels and involves different mechanisms.

One of the most significant mechanisms of drug resistance in cancer cells involves a notable reduction in intracellular drug concentration, which can occur due to decreased drug influx or increased drug efflux. As a result, the decreased drug concentration leads to reduced cytotoxicity of the drug, contributing to drug resistance in cancer treatment. The process of removing chemotherapeutic drugs from cancer cells involves the overexpression and activity of ATP-binding cassette (ABC) transporters, specifically ABCB1 (multidrug resistance protein or P-glycoprotein, MDR1/P-gp), ABCC1 (multidrug resistance-associated protein 1, MRP1), and ABCG2 (breast cancer resistance protein, BCRP) [102,103]. ABCB1 has broad substrate specificity with a large number of compounds identified as its substrates. ABCB1 regulates the efflux of various clinically important chemotherapeutics such as doxorubicin, paclitaxel, vincristine, and etoposide [104]. ABCC1 has a similar substrate specificity to ABCB1. However, ABCC1 exports substrates conjugated with glutathione (GSH). Thus, in addition to hydrophobic molecules, ABCC1 is also able to export organic anions [105]. ABCC1 extrudes many important cancer chemotherapeutics, including doxorubicin, vincristine, methotrexate, etoposide, and irinotecan [106]. ABCG2 also eliminates a wide range of chemotherapeutics, such as doxorubicin, methotrexate, and irinotecan [106].

Conversely, the changes in the mechanism by which anticancer drugs enter cancer cells lead to reduced drug influx. Copper uptake protein 1 (CTR1) plays an important role in the cellular uptake of cisplatin [107]. Consequently, CTR1 expression influences patient response to cisplatin therapy [108]. Methotrexate is predominantly taken up by cancer cells via the reduced folate carrier. Therefore, mutations in this carrier or its reduced expression lead to methotrexate resistance [109].

Drug inactivation mediated by the drug-metabolizing enzymes can reduce the amount of free drug available to bind to intracellular targets. An essential step in the inactivation of cisplatin is conjugation with GSH in a reaction mediated by the enzyme glutathione S-transferase. Consequently, higher expression and activity of glutathione S-transferase and increased intracellular GSH levels play a role in resistance to cisplatin [110,111].

Mutations in drug targets or changes in their expression levels have a significant role in developing drug resistance. Irinotecan is a potent inhibitor of DNA topoisomerase I (Topo I). Therefore, the downregulation of Topo I mRNA is correlated with resistance to irinotecan [112]. In addition, mutations in the Topo I gene impair the sensitivity of cancer cells to irinotecan [109]. Doxorubicin and etoposide target DNA topoisomerase II (Topo II). Like irinotecan, reduced Topo II levels or mutations in the Topo II gene have been associated with resistance to doxorubicin and etoposide [109]. The mitotic inhibitors paclitaxel and vincristine bind to the microtubules of the mitotic spindle and suppress their polymerization, preventing the regular course of cell division. Consequently, mutations in α- and β-tubulins, changes in the composition of β-tubulin isoforms, and alterations in microtubule dynamics influence resistance to antimicrotubule agents [113].

The ability of the cancer cell to repair DNA damage may determine sensitivity to anticancer drugs that induce DNA damage. Nucleotide excision repair (NER) is the main pathway to repair cisplatin-induced DNA damage [114]. In addition to increased DNA repair, increased tolerance to DNA damage also has a significant role in drug resistance. Various preclinical and clinical studies have shown that a lack of mismatch repair (MMR) genes confer resistance to cisplatin [114,115]. Additionally, a clinical study showed that loss of MMR is a predictive factor for response to irinotecan in colorectal cancer [116].

The tumor suppressor p53 is central in determining whether cells will arrest their growth or undergo cell death in response to chemotherapy. As a result, mutations in the TP53 gene that cause dysfunction in the p53 protein can reduce the sensitivity of cancer cells to chemotherapy [117]. Several studies have shown that loss of p53 function increases resistance to 5-FU [118]. In addition, several clinical studies have found that overexpression of TP53 protein product is associated with resistance to 5-FU chemotherapy [119,120]. Some clinical studies have also shown that ovarian tumors with mutations in TP53 have a poorer clinical outcome after cisplatin therapy [109]. Additionally, mutations in TP53 have been associated with a lack of response to doxorubicin in patients with breast cancer [121].

A precise mechanism for resistance to rituximab, a monoclonal antibody, is the loss of its target antigen CD20. This loss can result from reduced CD20 expression and deletions in the CD20 gene. In addition, another mechanism, known as “shaving”, can contribute to the loss of CD20 in rituximab-resistant malignancies. In this process, monocytes remove rituximab/CD20 complexes from the B-cell surface via the Fc receptor pathway [122]. Recently discovered molecular and cellular mechanisms behind trastuzumab resistance include immunosuppression induction, vascular mimicry, hypoxia, cancer stem cell emergence, HER2 heterogeneity and stability, alternative signaling pathways, and metabolic adaptation [123].

Patients who initially respond to immune checkpoint inhibitors may later develop resistance, leading to disease progression [124]. This resistance occurs because the tumor cells and tumor microenvironment (TME) undergo changes after interaction with the immune system, allowing the tumor to evade immune attack [125]. The mechanisms of acquired resistance to immune checkpoint inhibitors can be summarized as tumor-mediated acquired resistance, upregulation of inhibitory immune checkpoint expression levels, and increased immunosuppressive components in the TME [126].

The alterations in the target kinases, such as gene mutations or altered protein levels, are the main mechanisms of drug resistance to TKIs. The BCR-ABL inhibitor imatinib, the first FDA-approved TKI, is currently used as first-line therapy for CML. However, imatinib cannot entirely remove the BCR-ABL-expressing leukemic cell population, and resistance often occurs [127]. Different BCR–ABL–dependent mechanisms of imatinib resistance have been discovered, including amplification of the BCR-ABL oncogene, changes in BCR-ABL localization in CML cells, and mutations in the BCR-ABL oncogene. Point mutations can occur at different BCR-ABL sites, leading to relative or absolute resistance to imatinib [128]. This problem has led to the development and clinical use of second-generation BCR-ABL inhibitors, nilotinib, dasatinib, and bosutinib, and the third-generation BCR-ABL inhibitor, ponatinib. The BCR-ABL mutation T315I also confers resistance to all second-generation BCR-ABL inhibitors, but the response to ponatinib is preserved [129].

Most activating EGFR mutations in NSCLC patients are the exon 19 deletion and the exon 21 point mutation L858R. These mutations are managed with the first- and second-generation EGFR-TKIs: erlotinib, gefitinib, and afatinib. The primary mechanism of acquired resistance to first- and second-generation EGFR-TKIs is the substitution of threonine for methionine at position 790 in exon 20 (T790M) of the EGFR gene [130]. The third-generation EGFR TKI, osimertinib, selectively inhibits EGFR carrying all three common mutations, 19del, L858R, and T790M, while sparing wild-type EGFR [131]. Other known mechanisms of resistance to first- and second-generation EGFR TKIs include MET amplification, mutations in BRAF or PIK3CA, and amplification of genes encoding ERBB2 kinases [132].

It is a well-known phenomenon that some TKIs are substrates for ABC transporters. Therefore, ABC transporters can eliminate these TKIs from the cell, leading to resistance to TKIs. It is important to emphasize that some TKIs can act as both a substrate and an inhibitor of the ABC transporter, depending on the concentration range. Namely, imatinib is a substrate for both the ABCB1 and ABCG2 transporters, but only at a low concentration, while at higher concentrations, imatinib effectively inhibits the activity of these transporters [133]. The second-generation BCR-ABL inhibitors, nilotinib, and dasatinib, interact with the ABCB1 and ABCG2 transporters similarly to imatinib.

Interestingly, bosutinib inhibits the activity of ABCB1 and ABCG2 but does not act as a substrate for these transporters in the therapeutic concentration range [134]. The first-generation EGFR inhibitors, erlotinib, and gefitinib, also interact with ABCB1 and ABCG2 transporters. These EGFR inhibitors are actively transported by ABCB1 and ABCG2 at lower concentrations, while at higher concentrations, they can act as potent inhibitors of ABCB1 and ABCG2 function [135,136].

## 4. Functional Precision Medicine Platforms

**Figure 1:**
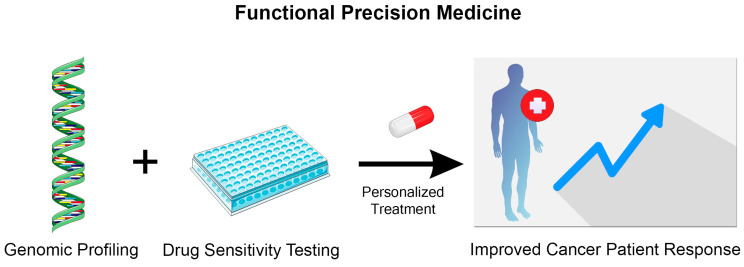
Functional precision medicine in cancer treatment. Functional precision medicine integrates genomic profiling with drug sensitivity testing of patient-derived cancer cells to identify personalized treatments and improve patient response to therapy. This Figure was created using images adapted from Servier Medical Art (Servier, smart.servier.com (accessed on 28 June 2024), licensed under a Creative Commons Attribution 3.0 Unported License).

FPM, which combines genomic profiling with sensitivity testing of patient-derived cancer cells (Figure 1), has the potential to identify effective treatments when clinically applied therapies fail [137,138]. FPM aims to tailor medical treatment to the individual characteristics of each patient by using different platforms to study the functional responses of patient-derived samples to different therapeutic interventions. These platforms include 2D cell cultures, 3D cell cultures, and PDX models, each offering unique advantages.

### 4.1. 2D Cell Culture Models in Functional Precision Medicine

**Figure 2:**
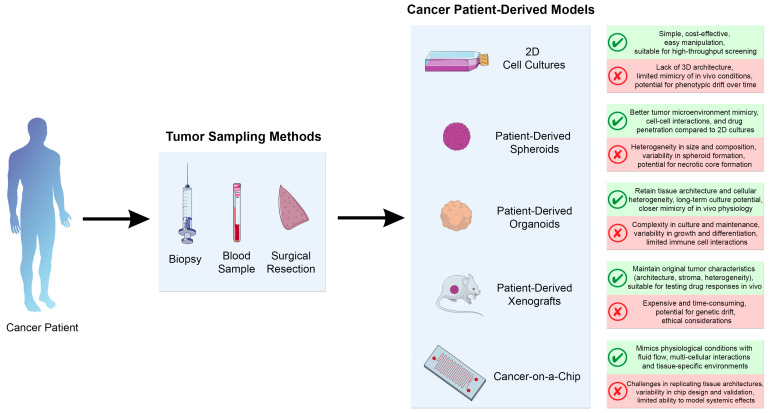
Platforms used in precision medicine. Cancer patient-derived models are developed from tumor samples collected through biopsy, blood samples, or surgical resection. The Figure illustrates different cancer patient-derived models used for studying functional responses to different therapeutics, including 2D cell cultures, spheroids, organoids, xenografts, and cancer-on-a-chip models. The figure also outlines the advantages and limitations of each model. This figure was created using images adapted from Servier Medical Art (Servier, smart.servier.com (accessed on 28 June 2024), licensed under a Creative Commons Attribution 3.0 Unported License).

Two-dimensional cell culture models play a crucial role in biomedical research, particularly within FPM platforms, where they provide valuable insights into personalized treatment strategies. By utilizing cells directly from the patient, it is possible to create personalized in vitro models that accurately reflect the genetic and phenotypic characteristics of the patient’s cancer (Figure 2). In the context of FPM, 2D cultures are utilized to analyze the functional responses of patient cells to various therapeutic agents, ultimately facilitating the development of tailored treatments for individual patients [139,140,141]. Patient-derived 2D cultures are particularly useful for FPM due to their simplicity and cost-effectiveness. In these models, cells are grown on flat, two-dimensional surfaces, making them easy to handle and maintain. This simplicity enables high-throughput screening of numerous compounds and facilitates rapid assessment of therapeutic efficacy and toxicity on patient-derived cells [139]. They are well suited for high-throughput drug screening and genetic studies, and the methods are highly standardized, which facilitates reproducibility. The primary sampling methods for patient-derived cancer cells include biopsies, blood samples, and surgical resections [142,143]. A biopsy involves taking a small tissue sample from the patient’s tumor with a needle or surgical instrument to obtain a direct source of cancer cells for culture and analysis. This method is critical to obtain cells that accurately reflect the genetic and phenotypic characteristics of the patient’s cancer. Blood samples are used to isolate circulating tumor cells or other relevant biomarkers from the patient’s bloodstream that provide insight into the cancer’s metastatic potential and its response to treatment. These samples are less invasive than tissue biopsies, making them a preferred option for serial monitoring of disease progression and treatment efficacy. In surgical resection, the tumor is removed during surgery. The removed tissue is used to create primary cell cultures that closely mimic the patient’s tumor environment. This method provides a large amount of tumor material for analysis and drug testing.

Recent studies have shown significant progress in the generation of primary cancer cells from patient tissue, particularly in the development of methods for isolating and culturing primary tumor cells from various malignancies. One study reported the successful generation of primary cancer cells from 568 patient samples, including core biopsies, fine needle aspirates, pleural effusions, and resections from a range of malignancies, including colon, lung, pancreatic, breast, endometrial, and head and neck cancers [142]. In a successful primary culture, the cancer cells no longer required an irradiated fibroblast feeder layer for growth, were free of stromal fibroblasts, could be cryopreserved, thawed, and re-grown, and had the same driver mutations as the original biopsy sample. The success rate in generating a cancer cell monoculture was 26% for all cancer types. The success rate was particularly high for lung cancer: 29% of the samples yielded a finished cancer cell line, compared to only 15% for breast cancer (*p* < 0.01) [142]. A comparative analysis of success rates in 61 lung cancer samples showed that the method using the fibroblast feeder layer and specifically defined tumor culture media had a higher success rate compared to standard media conditions.

Despite their advantages, 2D cell cultures have their limitations in FPM. The two-dimensional environment does not accurately replicate the three-dimensional architecture and interactions in living tissues. This lack of complexity can lead to discrepancies between in vitro drug responses and clinical outcomes. For this reason, FPM platforms often combine 2D cultures with more advanced models, such as 3D cultures and PDX, to enable a more comprehensive and physiologically relevant analysis.

### 4.2. 3D Cell Culture Models in Functional Precision Medicine

Patient-derived 3D cell culture models such as spheroids and organoids represent a significant advance in cancer research as they provide a more physiologically relevant environment by enabling the growth of cells in three dimensions and closely mimicking the structure and function of human tissue (Figure 2).

Spheroids are spherical aggregates of cancer cells or other cell types that naturally assemble in a suspension or low-adhesion environment [144]. They mimic the 3D architecture of tumors, including the nutrient, oxygen, and metabolic product gradient from the outside to the core. This gradient is critical for studying tumor biology and treatment responses because it mimics the conditions in solid tumors where the core often becomes necrotic due to hypoxia. Patient-derived spheroids have been generated from various cancers such as lung [145], gastric [146], colorectal [147], and breast cancer [148]. Organoids are miniaturized, simplified versions of organs produced in vitro from stem cells or organ-specific progenitor cells [144,149]. They are able to self-organize into structures that resemble the morphology and function of real organs. Similar to spheroids, patient-derived organoids (PDOs) accurately reflect the patient’s unique molecular signature and provide a precise model of tumor genetic and molecular diversity, hypoxia, nutrient diffusion, and metabolism [150]. The tumor samples used for PDO generation can be obtained from solid surgical resections, punch or fine needle aspiration biopsies, or biological fluid biopsies. PDOs have been successfully created from various types of cancer, including ovarian [151], breast [152], lung [153,154], gastrointestinal [155], esophageal [156], colorectal [157], prostate [158], liver [159], pancreatic [160], glioblastoma [161], retinoblastoma [162], and bladder cancers [163].

In a 2015 study, researchers established a living organoid biobank from colorectal cancer patients, demonstrating the capability of organoid cultures to generate high-quality drug sensitivity data and uncover gene-drug connections [164]. This platform successfully recapitulated clinical observations, such as cetuximab’s effectiveness in KRAS wild-type organoids and the responsiveness of TP53 wild-type organoids to Nutlin-3a. The identification of stem cell markers in patient-derived colorectal cancer organoids, such as clusterin, has led to the development of useful markers for drug resistance, patient survival, and disease recurrence [165]. Similarly, PDOs have shown promise in modeling treatment responses for metastatic gastrointestinal cancers, mirroring patient outcomes, and enabling personalized treatment strategies [155]. In pancreatic cancer, a library of 66 PDO cultures was obtained from fine-needle biopsies, surgical resections, and rapid autopsies collected from multiple clinical institutions [166]. The library allowed researchers to correlate PDOs’ drug sensitivity profiles with patient responses, providing chemosensitivity signatures that enable better treatment determination. Furthermore, the breast cancer PDO model has been suggested as suitable for predicting patient response to neoadjuvant chemotherapy [167]. Another study demonstrated the importance of intratumor heterogeneity in drug response using liver PDOs, showing that multiple cancer biopsies are crucial since organoids from different tumor slices exhibited varying mutations and responses to drugs [168].

The greater physiological relevance of spheroids and organoids compared to conventional 2D cultures results from their ability to better mimic the spatial organization and microenvironment of real tissue. This is important to study cellular processes such as proliferation, differentiation and apoptosis in a context that closely resembles in vivo conditions. The complexity of patient-derived spheroids and organoids leads to drug responses that are more predictive of in vivo outcomes and better capture the effects of drug penetration, resistance, and metabolism than 2D cultures. For example, the dense structure of the spheroid models restricts the penetration of drugs. Multiple cell layers act as barriers that can lead to conditions such as hypoxia and acidosis, which are associated with increased chemoresistance [169]. This makes them invaluable for preclinical drug testing and for reducing dropout rates in clinical trials. In addition, spheroids and organoids enable the study of complex cell–cell and cell–matrix interactions in the tissue microenvironment, which is critical for understanding tumor growth and metastasis [170].

However, the implementation of patient-derived 3D cultures is also associated with technical difficulties. The average success rate in generating PDOs for lung cancer is reported to be only 40%, often due to the quality of the biopsy sample [153]. In addition, the spatial heterogeneity of the original tumor cannot be fully captured in a single biopsy, so multiple PDOs from different tumor regions could be required to fully evaluate the effects of drugs [171,172]. In addition, scaling up 3D cultures for high-throughput screening is challenging due to the high cost of reagents, materials, and labor, and limited automated handling and analysis compared to 2D cultures. Despite their limitations, PDOs are crucial for progress in FPM. Ongoing development and optimization of 3D culture technologies to overcome the challenges posed by technical complexity and scalability is crucial to fully exploit their potential in cancer research and clinical applications. Recent advances in this area are promising, such as a new rapid protocol for producing PDOs and predicting drug response [153].

### 4.3. Patient-Derived Xenograft Models in Functional Precision Medicine

PDX models represent an innovative approach to cancer research and functional precision medicine, providing a platform to study tumor biology and drug response (Figure 2). PDX models are created by implanting fresh tumor tissue from cancer patients into immunocompromised mice [149,173,174]. This process maintains the intricate structure, genetic diversity, and cellular composition of the original human tumor, ensuring that PDX models highly reflect individualized patient cancers. The development of PDX models begins with the surgical resection or biopsy of a patient’s tumor [175]. The removed tumor tissue is usually fragmented into smaller pieces and implanted subcutaneously or orthotopically into the organ of origin of immunocompromised mice such as nude or NOD/SCID mice. Once the tumor has established itself in the mouse, it can be propagated over several generations by re-implanting tumor fragments into new recipient mice [176]. This procedure ensures a continuous supply of tumor tissue for experimental studies and preserves the properties of the original tumor over time. Successful PDX models have been developed from tumor tissue of various types of cancer, such as lung [145,177], breast [178], colorectal [179,180], bladder [181], and melanoma [182]. Circulating tumor cells have also been used to create PDX models for pancreatic [183], breast [184], and prostate cancer [185].

The establishment of patient-derived xenografts from non-small cell lung cancer patients demonstrated that these models maintain primary tumor features and correlate with patient prognosis and survival, suggesting their use in predicting lung cancer aggressiveness [186]. In another study, PDX models derived from 92 patients with various solid cancer types were screened against 129 treatments, showing that PDXs conserve the genetic patterns of primary tumors and accurately replicate patient responses to oncology therapies [187]. Furthermore, endometrial cancer PDX models revealed significant genetic similarities to primary tumors and highlighted the potential of combination therapies to stabilize tumor growth in pretreated patients [188]. In a recent study, PDX models were used for high-throughput drug screening and treatment determination for 56 high-risk pediatric cancer patients. They identified treatment options for 70% of patients, which led to a change in the treatment for 53% of patients, with 29% of those patients showing clinical benefits [189]. In pancreatic cancer, PDXs were utilized for pharmacokinetic/pharmacodynamic modeling and the characterization of dexamethasone’s effects [190].

PDX models have a range of applications in cancer research, drug development, and personalized medicine. They play an important role in preclinical drug testing and represent a valuable intermediate step between in vitro studies and clinical trials [154,187,191,192,193]. By testing drugs on PDX models derived from different patient tumors, the efficacy of therapeutic agents can be evaluated in different genetic backgrounds and tumor subtypes. PDX models are also used to study the mechanisms of drug resistance and to identify possible strategies for overcoming it [194,195,196,197]. By exposing PDX models to prolonged drug treatment, the genetic and molecular changes that lead to resistance can be investigated, as well as combination therapies that can prevent or reverse resistance [198].

PDX models are also crucial for biomarker discovery because comparing the molecular profiles of responding and non-responding PDX tumors can identify predictors of treatment response [199,200,201]. These biomarkers can then be validated in clinical trials and serve as a basis for treatment decisions in the clinic. The integration of PDX models with advanced omics technologies such as genomics, transcriptomics, proteomics, and metabolomics enhances their function by enabling high-throughput sequencing and other omics approaches to characterize PDX tumors, providing insights into their genetic and molecular profiles, identifying new therapeutic targets and pathways, and improving the precision of cancer treatment [201,202].

Recent advances in the development of PDX models include the creation of humanized mouse models [203]. These models are engrafted not only with patient-derived tumors but also with human immune cells, enabling the study of tumor-immune interactions and the evaluation of immunotherapies. Humanized PDX models are particularly valuable for the investigation of immune checkpoint inhibitors and other immunotherapeutics, as they provide insights into their mechanisms of action and potential clinical efficacy [204,205,206].

### 4.4. Cancer-on-a-Chip Models in Functional Precision Medicine

Patient-derived cancer-on-a-chip models are an innovative approach in the field of personalized cancer research and treatment [207]. Microfluidic technology is crucial for the development of patient-derived cancer-on-a-chip models. It uses tiny channels and chambers to precisely control the microenvironment and simulate conditions in human tissue (Figure 2). This technology advances cancer-on-a-chip models by enabling precise environmental control, high-throughput capabilities, and real-time analysis, making it essential for personalized cancer research and tailored therapies to improve patient outcomes [208,209,210,211,212,213,214,215]. By using cells taken directly from a patient’s tumor, these models provide a detailed representation of the unique genetic and phenotypic characteristics of an individual’s cancer. This level of personalization allows for more relevant and reliable predictions of how a patient’s cancer might respond to different treatments, increasing the potential for tailored treatment strategies [214]. The improved biological accuracy of these models is another important advantage. Unlike traditional 2D cell cultures, the cancer-on-a-chip systems replicate the 3D structure and TME, including critical cell-cell and cell-matrix interactions [216,217]. This capability leads to a better understanding of tumor behavior and drug response, making these models an invaluable tool for preclinical testing and drug development.

A cancer-on-a-chip platform allows extensive drug screening and has shown compelling correlations between ex vivo and clinical outcomes. For example, oxaliplatin-treated spheroids created from patients with the BRCA2 mutation in 3D-printed microfluidic devices demonstrated significant reductions in viability, reflecting the clinical sensitivity to platinum drugs [218]. Similarly, the glioblastoma-on-a-chip system was used to study the immunosuppressive microenvironments of glioblastoma [219]. This approach suggests co-targeting PD-1 immunotherapy with colony-stimulating factor 1 receptor (CSF-1R) inhibitors to enhance CD8+ T-cell functionality and induce apoptosis in glioblastoma. This patient-specific system provides personalized screening of immunotherapies, revealing distinct epigenetic and immune signatures in different glioblastoma subtypes. Furthermore, a 3D microfluidic chip for head and neck cancer patients evaluates the efficacy of immunotherapy by analyzing immune cell migration and cancer cell proliferation in response to immune checkpoint inhibitors [220]. This innovative humanized chip assay predicts the efficacy of immunotherapeutic drugs, tailoring treatments to individual patient profiles. These studies highlight the potential of cancer-on-a-chip models to revolutionize personalized medicine by providing accurate, patient-specific platforms for functional diagnostics and treatment optimization.

In addition, artificial intelligence can analyze data from patient-derived cancer-on-a-chip models to develop predictive models for cancer progression and treatment outcomes, identifying patterns that are not apparent in manual analysis. The development of automated systems for cell culture and analysis is critical to overcoming scaling issues and increasing the throughput of these models.

## 5. Functional Assays in Personalized Cancer Medicine

Functional assays in personalized cancer medicine offer several key advantages. First, they enable tailored treatment selection by allowing ex vivo modeling of a patient’s tumor from samples obtained during medical procedures. By analyzing the biological response of tumor cells to specific treatments, these assays provide a personalized functional profile that indicates chemosensitivity or resistance and helps to select the most effective treatment for individual cancer patients [6]. Second, they improve treatment outcomes by providing insight into how a patient’s tumor cells respond to different therapies, helping oncologists make more informed treatment recommendations. This personalized approach has the potential to improve patient response to therapy. Third, functional assays improve clinical decision-making by providing valuable information that can be integrated into the clinical process. By assessing the ex vivo response of tumor cells to different treatments, clinicians can make more precise and individualized treatment decisions. Fourth, these assays have shown that they can predict the efficacy of drugs or drug combinations on a patient’s tumor with high accuracy. Finally, studies have shown that functional assays can bring clinical benefits to patients by proving effective in different cancer types and improving treatment outcomes.

Various assays are commonly used for the functional analysis of 2D patient-derived cultures in FPM. Cell viability assays such as MTT and XTT measure metabolic activity to determine the health and proliferation of cells in response to drugs [221]. Cytotoxicity assays, such as LDH release and Annexin V staining, assess cell damage or apoptosis induced by therapeutic agents and provide information on the safety and efficacy of drugs. Gene expression analysis, usually by quantitative PCR or RNA sequencing, evaluates the effects of drugs on specific genetic pathways and helps to understand molecular mechanisms and identify treatment biomarkers [221]. Protein expression and post-translational modifications are analyzed using techniques such as ELISA or immunocytochemistry and provide information on the functional effects of drugs on cellular processes. High-content screening (HCS) is an advanced technique used in 2D cultures that combines automated microscopy with image analysis. HCS enables the simultaneous assessment of multiple cellular parameters, including cell morphology, intracellular signaling pathways, protein expression and subcellular localization of proteins [140,141]. This comprehensive analysis enables a detailed understanding of the effects of drugs on cell function, helping to select the most effective therapeutics for individual patients. Furthermore, immunoassays for HTC were developed to distinguish cancer cells from stromal cells using the CK8/18 antibody cocktail and to distinguish cells with a multidrug-resistant phenotype from sensitive cells using the ABCB1, ABCC1 and ABCG2 antibodies [140,141]. The simultaneous labeling of CK8/18 with the nuclear marker enabled a clear distinction and quantification of two different cell categories: CK8/18-negative cells, representing stromal cells, and CK8/18-positive carcinoma cells, allowing accurate quantification and analysis of each cell type within the mixed culture.

Three-dimensional cell assays for drug screening are applied to 3D culture models, including cancer-on-a-chip models, to better mimic the cellular environment in vivo and thus more accurately assess the efficacy and toxicity of drugs [193,222]. Assessment methods typically include techniques such as measuring ATP content to assess cell viability, the use of invasion chambers to assess cell invasion, the use of colorimetric or fluorescence-based assays for metabolic activity and viability, and the use of imaging techniques to visualize cell responses in a real-time [6]. Commonly used devices include luminometers, fluorescence microscopes, plate readers, and spectrophotometers. These assays play a critical role in studying various aspects of cell behavior, drug response, and tissue modeling in 3D cultures and provide a more physiologically relevant platform for drug screening and cell analysis compared to conventional 2D cultures. Although high-content imaging techniques enable detailed analysis of cell responses in 3D cultures, there are challenges in image acquisition, analysis, and visualization of the data. Multi-parametric analyzes help to normalize the data and examine the effects of treatment on specific cell subpopulations. Advanced tools are needed for software that enables true 3D phenotypic analysis and single-cell segmentation for high-throughput screening [222].

## 6. Limitations of Functional Precision Medicine Platforms

The phenotype of primary cells is significantly influenced by the isolation process and in vitro culture conditions, affecting cell morphology, proliferation, and expression of markers [223]. Various methods are used for isolating and expanding target cells in vitro, leading to differences in procedures and a lack of standardized protocols [224]. Enzymatic digestion affects primary cells depending on the type of enzymes used and treatment conditions, altering cell yield, viability, population, phenotype, and gene expression patterns [224]. Media formulations also play a critical role in influencing cell growth, viability, morphology, differentiation, gene expression, and overall functionality [225].

Studying TME in the context of primary cell cultures is very important. When developing advanced tumor cell-based models, the role of the stroma should not be overlooked [224]. Although it is challenging to incorporate all stromal cell types and subtypes into in vitro tumor models, certain cells are essential as they significantly influence the microenvironment. Another challenge is differentiating between primary cell subpopulations [224]. Conventional cell separation methods rely on cell type-specific markers, which may not adequately represent the diversity within cell types. Additionally, different studies use different markers to define the same cell types, leading to discrepancies in understanding cell behavior and function [224].

Two-dimensional primary cell cultures cannot replicate the natural architecture of tumors, resulting in an inaccurate representation of crucial cell–cell and cell–extracellular matrix interactions. Such interactions are essential for various cellular functions [226,227]. Disturbed interactions with the external environment cause adherent growing cells to lose their polarity, impairing their response to various external and internal stimuli [228]. After isolation and transfer to 2D conditions, cell morphology and division mode change, resulting in a loss of diverse phenotypes [227,228]. Morphological changes affect cell function, internal organization, secretion, and signal transduction [229].

In 2D cultures, the cells have unrestricted access to nutrients, oxygen, and signaling molecules, allowing faster growth [228]. They also receive an equal amount of anticancer drugs during treatments, unlike in vivo conditions [228]. Furthermore, the 2D culture system alters DNA methylation rates, gene expression, splicing, cell topology, and biochemistry, limiting the relevance of such models in mimicking in vivo conditions of cancer cells [230,231].

Both 2D and 3D primary cell cultures face significant limitations due to the unpredictable quality of human biopsies and surgical resections as a source of starting material. The generation of heterogeneous spheroid populations in terms of volume and shape poses a substantial challenge to the standardization of experimental protocols and interpretation of data [222]. Additionally, patient-derived spheroids may develop a central necrotic core as they increase in size due to restricted diffusion of nutrients and oxygen to the inner regions of the spheroid [232]. The presence of a necrotic core significantly reduces overall cell viability in the spheroid and poses a substantial challenge for drug penetration and efficacy testing [232]. Drugs may have limited access to the inner regions of the spheroid where the necrotic core is located, which significantly affects the response to treatment [232].

The time required to generate 2D and 3D primary cell cultures, including PDOs, can vary widely and can influence drug response studies [233]. The success of PDOs depends on tumor grade, cellularity of the resected tissue, early organoid formation after seeding, the composition of the culture medium, and control of fibroblast overgrowth [234,235,236,237].

Creating PDOs that faithfully replicate the heterogeneity of tumors is indeed challenging. PDOs do not fully represent the heterogeneity of the original tumor due to clonal selection during organoid establishment and represent only a subset of tumor cells that can miss important subpopulations [238,239]. Recent drug screening studies have reported intra-patient heterogeneity of drug response in PDOs derived from different cancer lesions of the same patient [166,237,240]. Although superior to 2D primary cell culture and 3D spheroids, PDOs can still deviate from the genetic profile of the original tumor, which may compromise their utility for drug screening and therapeutic development [239].

In addition, PDOs are limited in capturing the full complexity of the TME due to the absence of key stromal components [240]. PDOs do not include essential elements such as blood vessels, leading to slower material exchange compared to the natural process through blood vessels. This can impact both tumor growth and response to drugs [241]. The TME consists of various non-tumor cells, including cancer-associated fibroblasts, endothelial cells, and different types of immune cells [242]. These stromal cells play a crucial role in the tumor and engage with tumor cells through chemokines, growth factors, enzymes, and extracellular vesicles [243]. Consequently, PDOs are unable to accurately replicate the complex interactions between tumors and the immune system [239].

The process of establishing PDX models is not only time-consuming and expensive but also comes with its own set of challenges. It involves transplanting tumor tissue from patients into immunodeficient mice and then monitoring tumor growth and response to treatment. However, this process takes several months, making it unsuitable for real-time personalized medicine [244].

The time frame and resource-intensive nature of PDX model generation pose obstacles to its widespread use and feasibility for large-scale studies [245,246,247]. Additionally, the high failure rate for transplantation of certain cancer types and phenotypes remains a major obstacle to the widespread use of PDX models. Success in establishing PDX models varies depending on tumor type and characteristics, with highly malignant tumors having a higher likelihood of successful engraftment than less aggressive tumors.

PDX models may undergo genetic changes over successive passages that lead to genetic drift and divergence from the original patient tumor, affecting tumor response to therapies [248]. They also underrepresent subclonal heterogeneity, attributed to sampling bias due to spatial heterogeneity, differing capacities of subclones to engraft and proliferate, and tumor evolution and selection during PDX passages [249]. In addition, intratumoral heterogeneity can be influenced by external factors in the microenvironment, including murine host cells [250].

While offering significant advantages for cancer research, PDX models also pose challenges for data management and analysis. Sophisticated data integration techniques, normalization standards, and tailored analysis algorithms are necessary to ensure the reliability and accuracy of results [251]. Moreover, the use of animals in research, including the generation of PDX models, raises ethical considerations regarding animal welfare and the 3Rs principles (Replacement, Reduction, and Refinement) [251].

As an alternative to animal models, cancer-on-a-chip models have been extensively developed over the last ten years as the most challenging to implement 3D models [252]. The methods for establishing and culturing cells in cancer-on-a-chip models are often created individually by users without standardized guidelines, leading to variations in both technological and biological aspects. As a result, the lack of standardization impedes the scalability of results from the microscale to potential macroscale applications [253,254].

From a technological standpoint, performing multiple processes, such as biomarker separation, detection, analysis, and information retrieval on a single chip, is challenging. Further refinements are needed to generate precise gradient flows and shear stresses to reproduce conditions in vivo accurately [254]. Another limitation of cancer-on-chip devices is that they typically only replicate essential components and disregard important chemical and physical properties of the tumor microenvironment [255].

Moreover, polydimethylsiloxane (PDMS), which is commonly used for organ-on-chip production due to its biocompatibility, transparency, and oxygen permeability, has the drawback of non-specific absorption of small hydrophobic molecules, including some anticancer drugs. New materials need to be developed to retain PDMS’s properties while minimizing drug absorption [254,256].

## 7. Future Perspectives

After more than 50 years of cancer research following the official declaration of the war on cancer by President Richard Nixon of the United States [257], significant progress has been made, but many challenges remain. Despite hopes placed in precision medicine and genome-targeted approaches, the overall effectiveness of identifying actionable targets through molecular profiling in unselected cancer patients has been limited [13]. As a result, the routine clinical use of multigene NGS testing has been restricted [258]. The future goal is to identify more actionable molecular targets and understand how to target complex molecular interconnections to treat cancer patients more effectively. Artificial intelligence and analysis of existing data could play a crucial role in achieving this.

The ultimate focus should be on improving the overall survival and quality of life for cancer patients. While many targeted anticancer drugs have been approved in the past two decades, understanding their appropriate use for specific patients and obtaining data on overall survival is still a work in progress [259]. Although not all cancer patients have actionable targets, other genomic changes can indicate the use of available targeted therapeutics. In the future, functional drug screenings and multigene NGS testing should be considered to guide clinical decisions.

Understanding the genetic makeup of relapsed diseases is crucial for customizing effective treatments to combat drug resistance. Ongoing research aims to pinpoint genetic markers that can predict how a patient will respond to immunotherapy [260]. Studies have revealed that the presence of diverse genetic variations within tumors is associated with worse clinical outcomes across different types of cancer. Identifying these variations at a subclonal level is essential for predicting tumor regrowth or advancement [261]. By combining data from proteomic, metabolomic, epigenetic, and microRNA-based analyses, we can better tackle the complexities of cancer. Utilizing proteogenomic approaches can help connect genetic changes to observable traits, uncovering important cancer-related developments that may not be apparent through standard genetic analysis [262].

However, there are limitations associated with functional precision medicine platforms, including the inability to identify treatment-resistant cells, differentiate between cancer cell subpopulations, sampling bias, molecular drift during primary culture, lack of representation of TME, and complex molecular data interpretation. The most challenging aspect is the lack of standardization and scalability. Despite this, these imperfections might be acceptable if empirical evidence confirms the benefits for cancer patients. While there is a long road ahead, it is encouraging that many major academic centers all over the world are opting for early and comprehensive tumor and germline genetic testing for a large proportion of cancer patients [263]. This suggests that functional drug screening may soon be implemented in clinical practice to support a genome-targeted approach in cancer patients’ treatment.

## Figures and Tables

**Table 1:** The effectiveness of targeted therapies for cancer patients eligible based on identified biomarkers.

Tumor Type	Biomarker	Eligible Cancer Patients	Targeted Therapy and Corresponding Clinical Trials	Response Rate of Eligible Patients
Chronic myeloid leukemia (CML)	BCR-ABL fusion	>90% [14]	**Imatinib** approved in 2006 by the FDA IRIS clinical trial Improved PFS compared to IFN/Ara-C [15]	No data reported
			**Dasatinib** approved in 2006 by the FDA DASISION clinical trial Higher and faster rates of response compared to imatinib [16]	No data reported
Chronic lymphocytic leukemia (CLL)	Bruton tyrosine kinase	No data reported	**Ibrutinib** approved in 2014 by the FDA CLL12 clinical trial Better PFS than placebo in early-stage CLL Cardiotoxicity issues—not approved [17]	No data reported
			**Acalabrutinib** approved in 2017 by the FDA ASCEND clinical trial Improved PFS with a tolerable safety profile in these patients [18]	No data reported
NSCLC	EGFR	14.1–38.4% [19]	**Erlotinib** approved in 2013 by the FDA BR.21 clinical trial First-line and second-line therapy Better response in patients with EGFR overexpression [20] EURTAC clinical trial and OPTIMAL clinical trial Increased PFS compared to standard chemotherapy [21,22] RADIANT clinical trial Increased PFS but without a final conclusion [23]	77% * [13]
			**Gefitinib** approved in 2005 by the FDA WJTOG3405 clinical trial Increased PFS compared to standard chemotherapy [78]	
			**Afatinib** approved in 2013 by the FDA LUX-Lung 3 clinical trial Increased PFS compared to standard chemotherapy [24] Metadata study Increased PFS for major uncommon EGFR mutations (G719X, L861Q, and S768I, excluding T790M or exon 20 insertions) [25]	
			**Dacomitinib** Approved in 2018 by the FDA ARCHER clinical trial Increased PFS compared to gefitinib [26,27]	
			**Osimertinib** Approved in 2015 by the FDA UNICORN clinical trial Increased PFS for uncommon EGFR mutations [28] FLAURA 2 clinical trial Increased PFS and ORR in combination with chemotherapy than when applied alone [29] ADAURA clinical trial Increased PFS for all EGFR mutations compared to placebo [30]	
	ALK	2–7% [32,33]	**Crizotinib** approved in 2011 by the FDA PROFILE 1014 clinical trial Increased PFS compared to standard pemetrexed-plus-platinum chemotherapy [31]	79% * [13]
			**Alectinib** approved in 2015 by the FDA ALEX clinical trial Increased PFS compared to crizotinib with fewer side effects [34] ALINA clinical trial Increased PFS compared to platinum-based chemotherapy [35]	
			**Lorlatinib** approved in 2018 by the FDA CROWN clinical trial Increased PFS compared to crizotinib but with more adverse effects [36]	
	ROS1	0.9–2.6% [37,38]	**Crizotinib** approved in 2016 by the FDA EUCROSS clinical trial Increased PFS and ORR; worse response with TP53 co-mutation [39]	66% * [13]
	RET	1–2% [40,41,79]	**Selpercatinib** approved in 2020 by the FDA LIBRETTO-001 clinical trial Durable efficacy after treatment with platinum-based chemotherapy and alone; showed intracranial activity [42]	64% * [13]
			**Pralsetinib** approved in 2020 by the FDA ARROW clinical trial Response, regardless of previous therapy; showed intracranial activity [43]	
	MET	MET overexpression 15–70% [44] MET amplification 0.7–21% [45] MET Exon 14 Alterations 2–4% [46,47]	**Capmatinib** Approved in 2020 by the FDA GEOMETRY mono-1 clinical trial (MET exon 14 skipping mutation/MET amplification) Improved PFS in previously untreated patients compared to treated [80]	68% * [13]
			**Tepotinib** approved in 2024 by the FDA VISION clinical trial (MET exon 14 skipping mutation/MET amplification) Rapid and durable in patients whose MET alterations were identified by either solid or liquid biopsies [48]	
	VEGFR	No data reported	**Bevacizumab** approved in 2004 by the FDA AVAil clinical trial Increased PFS and ORR in combination with cisplatin/gemcitabine compared to chemotherapy alone [49]	No data reported
			**Sorafenib** approved in 2005 by the FDA NCT00449033 clinical trial No improvement in combination with cisplatin/gemcitabine compared to chemotherapy alone [50]	
			**Ramucirumab** approved in 2020 by the FDA REVEL clinical trial Improved PFS and OS in combination with docetaxel compared to docetaxel alone [51]	
	BRAF	2–5% [52,53]	**Vemurafenib** approved in 2011 by the FDA VE-BASKET clinical trial Durable response in BRAF V600-mutant NSCLC [54]	63% * [13]
			**Dabrafenib** approved in 2013 by the FDA NCT01336634 clinical trial Durable response in combination with trametinib with a manageable safety profile in BRAF V600E-mutant NSCLC, regardless of prior treatment [55]	
	PD-L1	≥1–53–63% [56,57,81]	**Nivolumab** approved in 2017 by the FDA CheckMate 816 clinical trial Increased PFS and ORR in neoadjuvant treatment with platinum-based chemotherapy [58]	No data reported
			**Pembrolizumab** approved in 2020 by the FDA KEYNOTE-024 clinical trial (PD-L1 score ≥50%) Increased OS compared to platinum-based chemotherapy [59]	
Breast cancer	EGFR	2.6–11.4% [60]	**Erlotinib** emerging evidence, not yet approved NCT00024219 clinical trial Minimal activity in unselected previously treated advanced-stage breast cancer [82]	No data reported
			**Afatinib** emerging evidence, not yet approved Lux-Breast 3 clinical trial Similar benefits compared to other treatments but with frequent adverse events and low tolerance [61]	
	EGFR/HER2	No data reported	**Lapatinib** approved in 2007 by the FDA NCT00073528 clinical trial (HER2+ and HR+ metastatic breast cancer) In combination with letrozole significantly enhances PFS and ORR [62]	No data reported
	HER2	2–5% [63,64]	**Trastuzumab deruxtecan** approved in 2019 by the FDA DESTINY-Breast04 clinical trial (HER2-low metastatic breast cancer) Increased PFS and OS compared to chemotherapy [65]	60% * [13]
	CDK4/6	ER+/HER2− ≈ 60% [66]	**Ribociclib** approved in 2017 by the FDA MONALEESA-2 clinical trial (HER2-negative advanced breast cancer) In combination with letrozole significantly improved PFS compared to letrozole alone [67]	No data reported
			**Palbociclib** approved 2015 by FDA PALOMA-2 clinical trial (HR+/HER2− breast cancer) palbociclib plus letrozole demonstrated consistent PFS gains versus placebo plus letrozole [68]	
Gastrointestinal stromal tumors	VEGFR	No data reported	**Bevacizumab** approved in 2004 by the FDA In combination with irinotecan-fluorouracil-leucovorin chemotherapy significantly improved PFS and OS in metastatic colorectal cancer [69]	No data reported
Urothelial carcinoma	PD-L1	15–44% [70,71,72]	**Avelumab** approved 2020 by FDA JAVELIN Bladder 100 clinical trial (PD-L1–positive and negative population) Improved OS and following platinum-based chemotherapy regardless of PD-L1 score [73]	No data reported
Melanoma	BRAF	43–66% [74,75]	**Dabrafenib** approved in 2013 by the FDA BREAK-2 clinical trial (BRAFV600E/K mut+ metastatic melanoma) Well tolerated and clinically active [76]	70% * [13]
	MAPK	No data reported	**Trametinib** approved in 2013 by the FDA METRIC clinical trial (BRAFV600E/K mut+ metastatic melanoma) Improved PFS and OS [83] NCT02296112 clinical trial (non-V600 BRAF mutations/BRAF fusions) Considerable clinical activity [77]	No data reported

* Estimates of absolute response rate in USA patients in 2020 [13].
